# Carbon Nanotube/Graphene Nanoplatelet Hybrid Film as a Flexible Multifunctional Sensor

**DOI:** 10.3390/s19020317

**Published:** 2019-01-14

**Authors:** JianRen Huang, XiaoXiang Yang, Shiuh-Chuan Her, Yuan-Ming Liang

**Affiliations:** 1 School of Mechanical Engineering and Automation, Fuzhou University, Fuzhou 350108, China; chinafzhjr@gmail.com; 2 Department of Mechanical Engineering, Yuan Ze University, Chung-Li 320, Taiwan; cliff0857@gmail.com; 3 Quanzhou Normal University, Quanzhou 362000, China

**Keywords:** carbon nanotubes, graphene nanoplatelet, piezoresistive effect, multifunctional sensor

## Abstract

A flexible hybrid film consisting of graphene nanoplatelets (GNPs) and multi-walled carbon nanotubes (MWCNTs) was prepared and employed as a multifunctional sensor to monitor temperature and liquid leakage, based on the piezoresistive effect. The influences of the GNP content on the mechanical, thermal, and sensing properties were investigated. Experimental results showed that both the hardness and Young’s modulus of the hybrid film were decreased with an increasing GNP content, while the thermal conductivity exhibited in an opposite trend. The electrical resistance of the hybrid film decreased was linearly with an increase in temperature. The resistance change increased linearly with an increase of the solvent adsorption. These features demonstrated the potential applications of the hybrid film in the detection of temperature, and liquid leakage. The sensitivity of leakage detection is increasing with the increase of the GNP loading, while temperature sensitivity is in the opposite trend.

## 1. Introduction

Carbon-based nanomaterials, including carbon nanotube (CNT) and graphene, have received increasing attention, owing to their excellent thermal, mechanical, chemical, and electrical properties [1,2]. Thin film or paper-like composite materials, consisting of multi-walled carbon nanotubes (MWCNTs) and graphene nanoplatelets (GNPs), exhibit high electrical and thermal conductivities [3], extraordinary structural flexibility, excellent mechanical properties [4], and a porous structure, with a high specific surface area [5]. Therefore, such hybrid films have the potential for application in strain sensors [6], batteries [7], gas or solvent sensors [8,9], flexible temperature sensors [10], and electromagnetic shielding [11]. In these freestanding hybrid films, CNTs and GNPs are cohesively bound by the van der Waals forces, π-π bond interactions, and mechanical entanglement. The thin films are easily fabricated through self-assembly by vacuum filtration [12].

CNTs and GNPs are applicable for sensors and have been of great interest to researchers. Rein et al. [13] employed a flexible CNT film strain sensor to monitor the deformation and damage of epoxy resins. Moriche et al. [14] investigated the piezoresistive performance of a strain-sensitive GNP/epoxy matrix. Davaji et al. [15] reported a suspended architecture composed of single-layer graphene on a substrate for application as a temperature sensor. Lu et al. [16] utilized a CNT film as a new measurement method to determine the glass transition temperature of polymeric composites, thus, providing a new way to understanding the glass transition phenomenon in composites. Xue et al. [17] developed a flexible polyaniline/CNT nanocomposite, assembled as a highly sensitive ammonia sensor, which exhibited highly sensitive NH3 sensing, with fast response/recovery times. Zhang et al. [18] reported a chemically reduced graphene oxide (RGO)/poly (diallylimethyammonium chloride) (PDDA) nanocomposite film sensor with high-performance humidity properties. Lin et al. [19] proposed a novel humidity sensor using graphene/TiO2 material by a sol–gel method. The humidity-sensing properties were characterized based on an inductance–capacitance–resistance analyzer. Chen et al. [20] utilized lignosulfonate (LS) as moisture sensing layers and reduced graphene oxide (rGO) as resistant transduction layers, to fabricate an rGO/LS composite thin-film as a humidity sensor, for a broad relative humidity ranging from 22% to 97%. Lee et al. [21] developed a micro temperature sensor using the physical vapor deposition (PVD) technique to monitor the temperature inside lithium-ion batteries. Yang et al. [22] presented a flexible temperature sensor, based on graphene nanowalls. Mahadeva et al. [23] reported a flexible humidity and temperature sensor, based on a cellulose-polypyrrole nanocomposite.

As mentioned above, most of the existing studies focused on the sensing capability of carbon nanomaterials, such as MWCNTs and GNPs, individually, and only few studies have reported on pore size distribution and thermal and micromechanical properties of hybrid films. This work seeks to explore the application of hybrid films as a temperature sensor and provides a reproducible and inexpensive qualitative analytical tool for liquid leakage detection. In this study, hybrid films with varied MWCNT-to-GNP weight ratios were fabricated by the vacuum filtration method, and the effect of the GNP content on the porous structure, thermal conductivity, mechanical properties, and sensing performance toward a temperature and liquid leakage was investigated. A controllable sensitivity of the hybrid film could be achieved by varying the GNP content. Our ultimate goal was to investigate the MWCNT/GNP hybrid films to further improve their properties for potential applications.

## 2. Fabrication of Hybrid Films

### 2.1. Materials 

**Figure 1:**
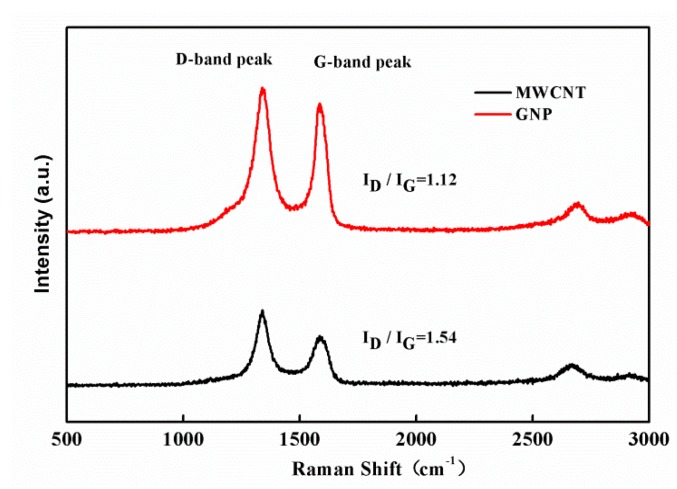
Raman spectrum of the graphene nanoplatelets (GNPs) and the multi-walled carbon nanotubes (MWCNTs).

In this work, MWCNTs prepared by the CVD method (length: 0.5–20 μm, thickness: 1–10 nm; Uchees Co., Taipei, Taiwan) and GNPs (diameter: 10–50 nm, length: 100–200 μm, carbon content: >98.5%; Conjutek Co., Taipei, Taiwan), were used to fabricate the hybrid films with different GNP weight ratios. Raman spectrum were obtained by a Raman spectrometer (Invia Reflex, Renishaw, UK), using a 532 nm laser source to evaluate the structure and quality of the MWCNT and GNP as shown in Figure 1. The D and G band peaks were the two most prominent characteristic peaks which represent the disordered sp^3^ structure and the sp^2^ hybridized graphitic structure, respectively. The intensity ratio of D band and G band can be calculated to characterize the quality of graphitic crystalline structures. The I_D_/I_G_ of GNP and MWCNT are 1.12 and 1.54, respectively. This indicates that GNP is better than that of MWCNT, in terms of the crystalline structure.

### 2.2. Film Preparation 

**Figure 2:**
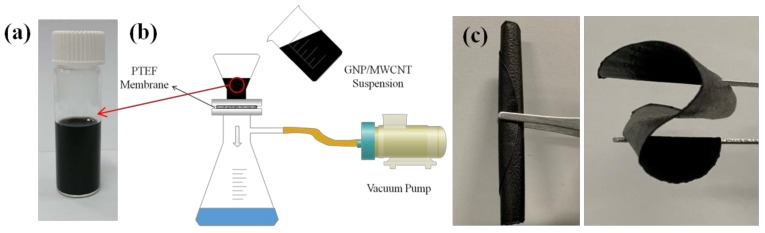
(**a**) MWCNT/GNP hybrid suspension, (**b**) schematic diagram of vacuum filtration, and (**c**) a flexible GNP/MWCNT hybrid film.

**Figure 3:**
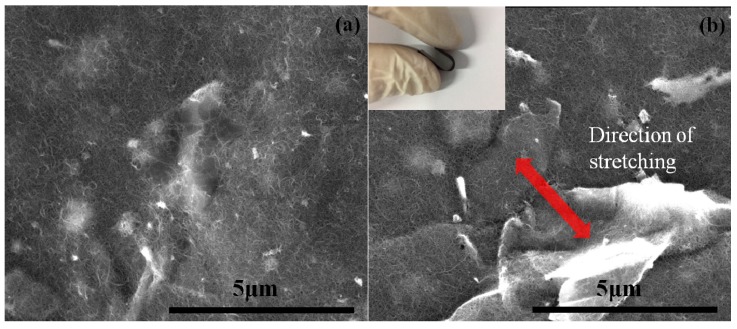
SEM images of the hybrid film GNP-50, (**a**) before and (**b**) after bending.

In this study, the film preparation method proposed by Hwang et al. [24] was adopted and has been briefly described. A total mass of 160 mg of GNPs and MWCNTs was mixed in a 300 mL aqueous (deionized water), with surfactant Triton X-100 (concentration of 0.01 g/mL), and sonicated (Qsonica Q700) in a pulse mode (on for 10 s, off for 20 s), at 30 W for 4 h. MWCNT and GNP were hydrophilic, due to the formation of functional groups, and were supported by Tritonx-100 [25,26]. Thus, nanoparticles could be well dispersed in water, by sonication, and self-assembly occurred through π–π stacking interactions, creating a compact 3D architecture, as a result of the steric hindrance effect of the graphene sheets [27]. Upon completion of the dispersion process, GNPs and MWCNTs were uniformly dispersed in the solution, and no precipitate was found, even after one month, as shown in Figure 2a. The MWCNT/GNP suspension was vacuum-filtered through a Polytetrafluoroethylene (PTFE) microporous filter membrane (pore size: 0.45 μm, diameter: 90 mm), as shown in Figure 2b. After filtration, the hybrid membrane was peeled off from the filter membrane, washed with isopropyl alcohol to remove the residual surfactant and impurities, and kept in a vacuum oven, at 40 °C, for 12 h, to obtain a freestanding film. Figure 2c shows the film is highly flexible, which can be rolled up various flexural deformations. Figure 3a,b illustrate the SEM images of the hybrid film GNP-50, before bending and after bending, respectively. It appears that no damage or fracture was observed on the hybrid film, after the bending.

In this study, a series of hybrid films, with different GNP weight percentages, were prepared, to investigate the effects of GNP on the mechanical and thermal properties. The GNP weight percentages were 0%, 10%, 20%, 25%, 30%, 40%, and 50% and the corresponding hybrid films were denoted as GNP-0, GNP-10, GNP-20, GNP-25, GNP-30, GNP-40 and GNP-50, respectively.

## 3. Characterization of the Hybrid Films

### 3.1. Morphology 

**Figure 4:**
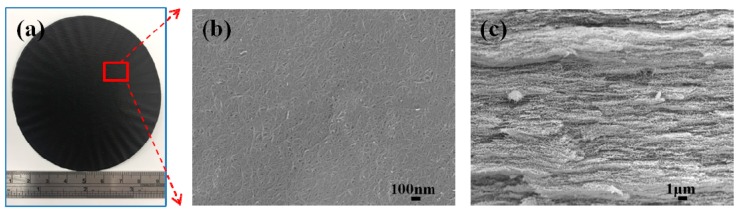
GNP-0 film with 0 wt% graphene and 100 wt% MWCNT; (**a**) the prepared film, (**b**) surface morphology SEM image, and (**c**) cross-section SEM image.

The microstructures of the hybrid films were characterized using a field emission scanning electron microscope (JSM-7600F, Jeol Ltd., Tokyo, Japan), and the samples were sputter-coated with a conductive gold layer, before obtaining the images. As shown in Figure 4a, a flexible film with a diameter of 80 mm and thickness of approximately 70 μm, was obtained. The hybrid film exhibits a metallic luster as the GNP content increases. This can be indicative of the intrinsic semiconducting behavior of the graphene [28].

**Figure 5:**
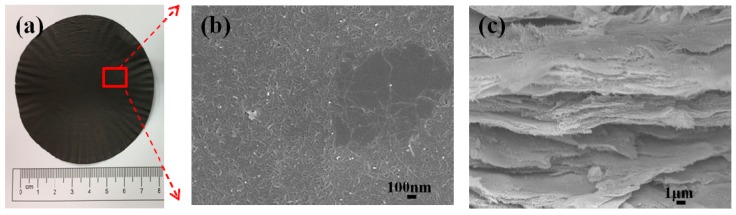
GNP-50 film with 50 wt% graphene and 50 wt% MWCNT; (**a**) the prepared film, (**b**) surface morphology SEM image, and (**c**) cross-section SEM image.

The surface morphologies and cross-section views of the hybrid film with 0 wt% (GNP-0) and 50 wt% (GNP-50) of the GNPs are presented in Figure 4 and Figure 5, respectively. Figure 4b clearly shows that MWCNTs were randomly oriented, and the long and tortuous MWCNTs were entangled with each other, forming a dense percolating network, and were homogeneously dispersed for the GNP-0 (0 wt% GNP and 100 wt% MWCNT) film, without any visible agglomeration. Some of the GNPs could be observed on the surface of the GNP-50 film (50 wt% GNP and 50 wt% MWCNT), and the GNP layers could conformably spread on the voids of the CNT network, as shown in Figure 5b.

**Figure 6:**
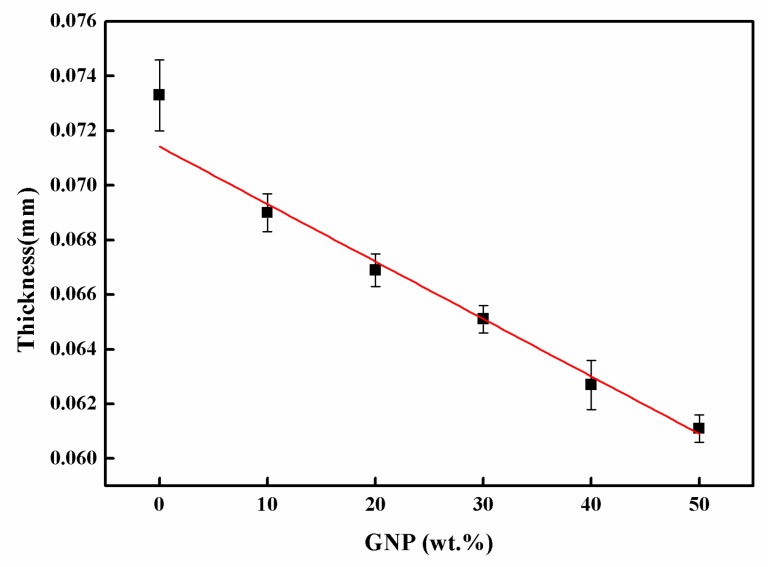
Thickness of hybrid film varied with the GNP weight percentages.

Figure 4c and Figure 5c show the cross-section of the hybrid films. MWCNTs and GNPs are successfully deposited to form a densely packed film with a “sandwich”-like structure, which can be attributed to the filtration-induced directional flow, during the fabrication process. The 3D MWCNT/GNP architecture was formed via self-assembly. With an increase in the GNP content, the structure of the hybrid film became gradually better aligned, in the horizontal direction, with an improved stacking behavior, and an increasingly thinner film was produced [29]. The thickness of the hybrid film decreased with the increase of the GNP content, as shown in Figure 6. 

### 3.2. Pore Size Distribution

**Figure 7:**
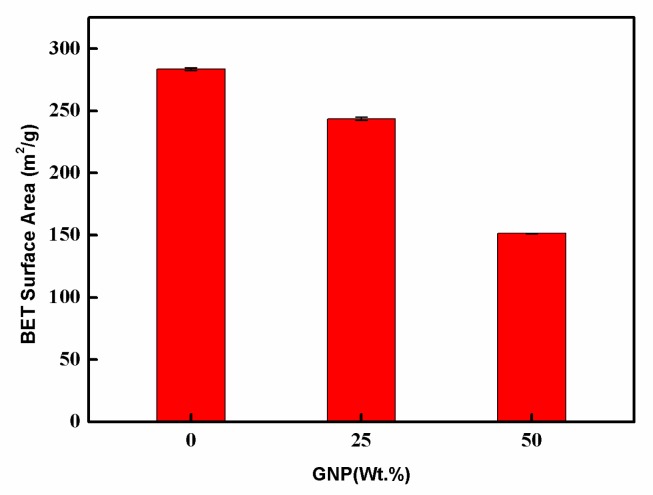
Surface area of the hybrid film varied with the GNP weight percentages.

**Figure 8:**
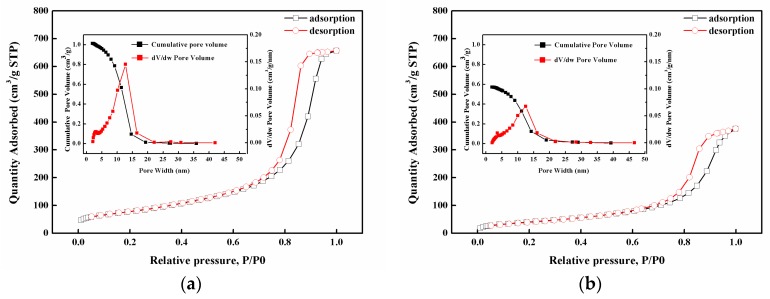
N_2_ absorption–desorption isotherms. Insets show the BJH pore size distribution. (**a**) GNP-0 with 0 wt% GNP and 100 wt% MWCNT and (**b**) GNP-50 with 50 wt% GNP and 50 wt% MWCNT.

The MWCNT/GNP hybrid film had a well-defined porous sandwich structure. To confirm the effect of the GNP on the creation of a porous structure, the surface area was measured by Brunauer-Emmett-Teller (BET) theory, as shown in Figure 7. The pore size distribution, using the Barrett-Joyner-Halenda (BJH) method based on nitrogen adsorption–desorption (ASAP2460, Micromeritics Corp., Norcross, GA, USA), is presented in Figure 8. The BET surface area decreased from 283.53 m^2^/g to 151.31 m^2^/g, as the GNP content increased from 0 to 50 wt.%. The pore distribution of the hybrid film was in the range of 5–25 nm. As expected, the total pore volume consistently decreased with an increasing GNP content.

The planar nature of GNPs enabled a compact packing, compared to the MWCNTs, which resulted in a lower porosity [1]. This is because the 2D GNP sheets tended to self-adjust their basal planes parallel to the plane of the filter membrane, during the vacuum filtration, to create a well-defined porous sandwich structure, yielding in a significant alignment of the GNP sheets. GNP could conformably spread on the voids of the MWCNT network, thus, reducing the porous structure formed by the MWCNTs. The compact, aligned structure reduced the surface area and pore volume, and led to the formation of a denser, electrically and thermally conductive, network.

### 3.3. Mechanical Properties

With a high-resolution of load and displacement, the nanoindentation test is a very useful technique for determining the mechanical properties of thin films [30,31]. A nanoindentation test system (Micro Materials, Wrexham, UK) was used to investigate the hardness and Young’s modulus of the hybrid films, with different GNP contents, at room temperature.

**Figure 9:**
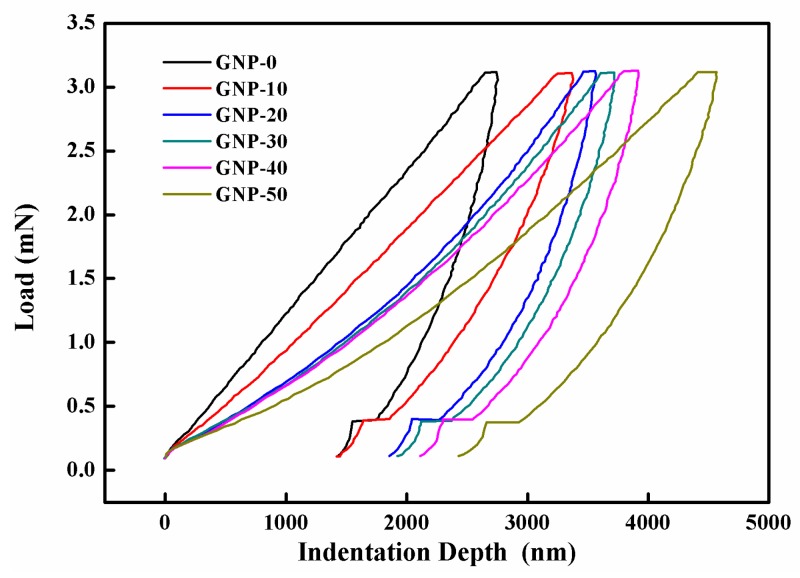
Load versus indentation depth curves for the hybrid films with different weight percentages of GNP.

In the nanoindentation tests, the Berkovich indenter was impressed into the hybrid film surface, under an increasing load, at a constant speed of 0.3 mN/s. After it reached a pre-determined maximum load of 3 mN, the sample was held under the peak force for 10 s, to minimize the time-dependent plastic effect. For each film, at least three points were measured. The representative load–indentation depth curves of the hybrid films, with different GNP contents, are compared in Figure 9. The GNP-0 film shows the highest resistance to indentation force with the lowest maximum indentation depth. It can be observed that the load–indentation depth curves were shifted to the right (which implies a larger indentation depth for the same load), as the GNP content increased. Moreover, the unloading curves exhibited discontinuities and small steps, which indicated that some cracks formed during indentation [32].

**Figure 10:**
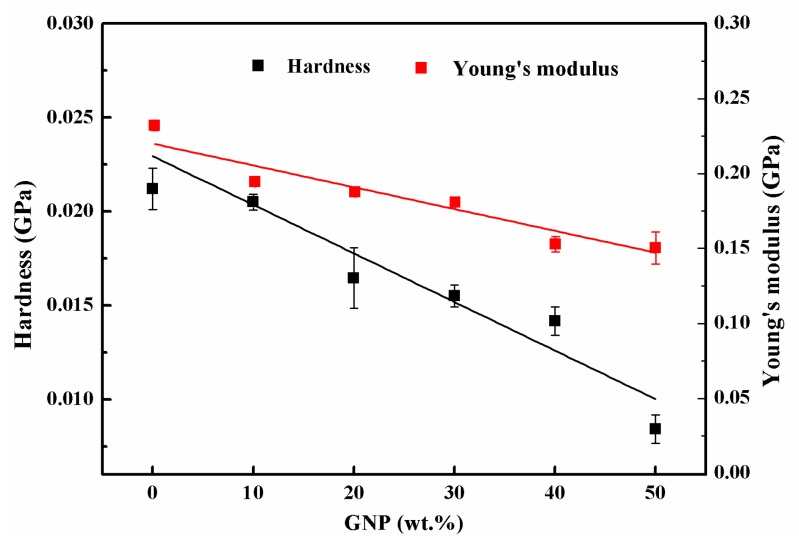
Hardness and Young’s modulus of hybrid films varied with the GNP weight percentages.

The hardness and Young’s modulus of the hybrid films extracted from the load–indentation depth curves are presented in Figure 10. It can be seen that both the hardness and Young’s modulus decreased with increasing GNP content. The hardness and Young’s modulus of GNP-50 decreased by 151.78% and 54.43%, compared to that of GNP-0, respectively. These results could be inferred from the cross-view SEM images, which revealed that the graphene sheets were mainly assembled by an in-plane contacting π–π interaction or van der Waals force, without any strong mechanical interlocking. However, the MWCNT bundles connected with each other through entanglement and formed a strong robust network. In addition, further increasing the graphene content led to a parallel decrease in the carbon nanotube axis directions, due to the excessive graphene loading, which deteriorated the structural integrity and destroyed the mechanical strength of the hybrid film [2].

### 3.4. Thermal Conductivity 

To explore the effect of the GNP content on the thermal conductivity of the hybrid film, the thermal conductivity (K) was calculated as follows [33]:

k(T) = α(T)∙Cp∙ρ(T)(1)

α(T) = 0.1388 × d^2^/t_50_ (2)

where, α, Cp, and ρ are the thermal diffusivity, heat capacity, and density, respectively. The thermal diffusivity was measured by a laser flash system (LFA457, Netzsch Group., Selb, GER), at room temperature. The prepared hybrid film was cut into a square strip (10 × 10 mm) for the measurement, d was the thickness of the sample and t_50_ was the half diffusion time. The heat capacity was measured using a differential scanning calorimeter (DSC214, Netzsch Group., Selb, GER), and the bulk density of the hybrid film was calculated from its weight and dimensions.

The measured thermal parameters of each sample are listed in Table 1 (room temperature). The thermal conductivity of the GNP-0 film was only 168.22 W/m∙k, while that of the GNP-50 increased up to 364.24 W/m∙k, mainly due to the increase in thermal diffusivity. The high thermal conductivity of the MWCNT/GNP hybrid film with 50 wt% of GNP was attributed to the 3D connected sheets, owing to the synergistic effect of the MWCNTs and GNPs. Most of MWCNTs, in the film were arranged in curled and wreathed forms, and the overlapping of the MWCNTs induced a larger interfacial thermal resistance that further decreased the thermal conductivity of the hybrid film. Furthermore, a large amount of gap existed between the MWCNT bundles, and the incorporated graphene, as a bridge overlapped with the dispersed MWCNTs, yielding a larger contact area than that of the MWCNTs. This promoted the transmission of phonons with thermal energy, which were more efficiently transported between the MWCNTs and GNPs, through the larger contact areas, thus, facilitating the heat transfer [34]. The prepared hybrid film consisted of alternately stacked GNPs and MWCNTs that imparted mechanical strength and thermal conductivity to the hybrid films, while the GNPs served as a highly conductive and heat-conducting component in the hybrid films.

## 4. Sensing Applications

Carbon nanomaterials such as graphene or CNT may exhibit semiconductor or metal properties. When graphene or CNT produced by the chemical method exhibits semiconducting properties, the resistance is determined by its thermally activated charge carriers. The mobility of the charge carriers is increased with an increase of the temperature, resulting in a decrease of the resistance. When graphene or CNT produced by the physical method exhibits metal properties, the resistance is determined by a charge carrier scattering. As the temperature increases, the carrier scattering is increased, leading to a decrease of the charge carrier mobility and an increase in resistance [10]. It was observed that the MWCNT/graphene hybrid film exhibited porous networks. Liquid interaction with the three-dimensional porous network might have altered the electrical conductance networks, resulting in a change of the electrical resistance. This inspired us to employ the MWCNT/graphene hybrid film as a multifunctional sensor, to detect both the temperature and a liquid leakage.

### 4.1. Temperature Sensor 

The prepared hybrid films were cut into rectangular strips (30 × 10 mm), to evaluate the temperature-sensing performance. The hybrid film sensor was attached to the center of a glass substrate, using thermal grease, to ensure a perfect bonding and heat transfer between the substrate and hybrid film sensor. Two electrodes were adhered to the hybrid film sensor, at a distance of 25 mm, using silver paste, to minimize the contact resistance. GNP and MWCNT were conductive enough to be used as electrodes and electrical contacts for resistance measurement [15]. The copper line was connected to a digital multimeter (Keithley 2450, Tektronix, Inc., Beaverton, OR, USA), for resistance measurement, while the temperature of the preheated vacuum oven was increased from 30 °C to 100 °C. The temperature was increased by 10 °C and then maintained constant, for 10 min, to ensure a thermal equilibrium between the test sample and substrates [35]. The resistance change of the hybrid film sensor and the temperature, measured by a thermocouple, were continuously recorded by a digital data acquisition system (cDAQ-9174, NI), through the LabVIEW software.

The temperature coefficient of resistance (TCR) is often used to describe the temperature-sensitive properties, popularly known as sensitivity [36].

TCR = (R − R_0_)/(R_0_∙∆T) (3)

where R, R_0_, and ∆T are the measured resistance, resistance at the initial temperature of 30 °C, and the change in temperature (°C), respectively.

**Figure 11:**
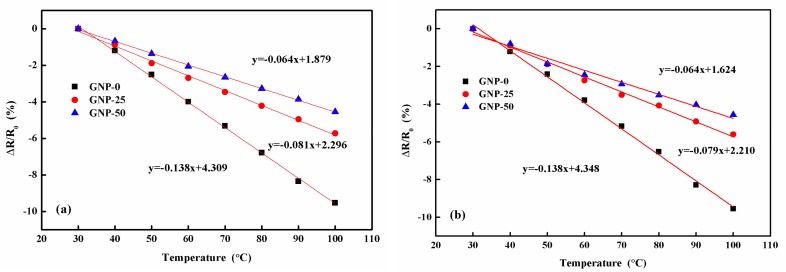
Normalized relative resistance change of the hybrid films varied with temperature. (**a**)~Atomsphere environment; (**b**) Vacuum oven.

Figure 11 plots the normalized relative resistance change versus temperature for the hybrid films, with different GNP contents in the atmosphere environment, with a relative humidity of 70%, and in a vacuum oven. It can be observed that the resistance vs. temperature curves in the atmosphere environment (Figure 11a) and a vacuum oven (Figure 11b) were the same. The resistance change was mainly attributed to the temperature change, while the ambient humidity did not affect the resistance of the hybrid film. The electrical resistance decreased with an increase in temperature, which indicated a negative piezoresistive behavior of the hybrid film. The TCR could be obtained from the slope of the curve. The values of the TCR for the hybrid films, with GNP contents of 0 wt%, 25 wt%, and 50 wt% were −0.1373%/°C, −0.0786%/°C, and −0.0644%/°C, respectively. The temperature sensitivity of the hybrid film decreased with the increase of the GNP content [37,38]. The TCR of the proposed hybrid film was similar to that of the MWCNT temperature sensor reported by Liu et al. [10], with a temperature sensitivity (TCR) of −0.068%/°C. 

The electrical resistance of the hybrid film could be classified into two different types of resistances—the intrinsic resistance of the nanomaterial [39] and the junction resistance (including contact resistance and tunneling resistance) [40]. In the neat MWCNT (GNP-0) film, the MWCNTs formed a network, with a large number of joints. Thus, the junction resistance became the dominant factor during the temperature change. The increase of the GNP content led to an increase in the layered structure of the film and a decrease of the joints in the network. It could be considered that the intrinsic resistance of the GNPs became the main resistance source of the hybrid film. Therefore, the relative resistance change in the GNP-50 was reduced.

**Figure 12:**
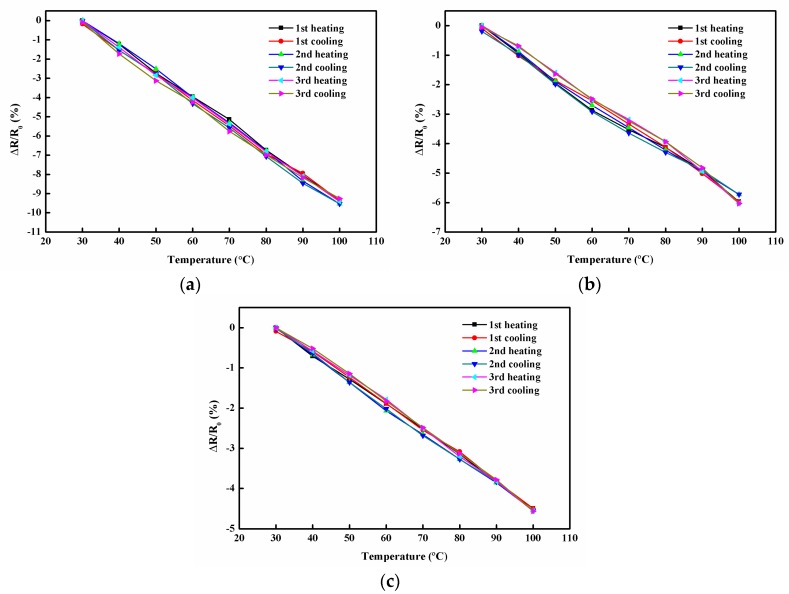
Normalized relative resistance change of the hybrid films subjected to cyclic heating and cooling tests. (**a**) GNP-0 with 0 wt% GNP and 100 wt% MWCNT. (**b**) GNP-25 with 25 wt% GNP and 75 wt% MWCNT. (**c**) GNP-50 with 50 wt% GNP and 50 wt% MWCNT.

Furthermore, to investigate the reliability and stability of the hybrid film as a temperature sensor, the hybrid films were subjected to cyclic heating and cooling tests, as illustrated in Figure 12. No obvious change was observed in the △R/R_0_ vs. temperature curves, which demonstrated the high stability and durability of the hybrid film for the temperature-sensing application.

**Figure 13:**
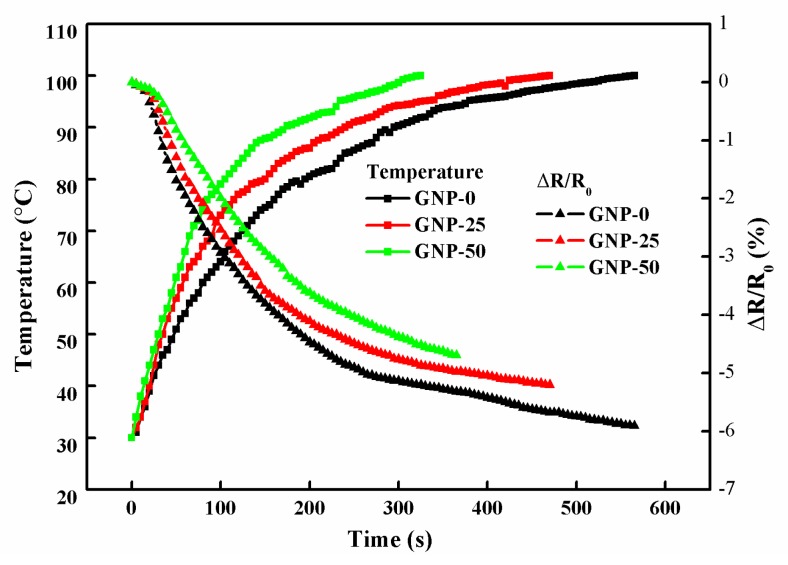
Temperature and resistance responses of the hybrid films placed in an oven at temperature of 100 °C.

Hybrid films with three different GNP weight percentages of 0 wt%, 25 wt%, and 50 wt% were placed in an oven, at 100 °C. Figure 13 illustrates the temperature and resistance responses of the hybrid films. It shows that the response time decreased with the increase of the GNP content. GNP-50 with 50 wt% of GNP exhibited the fast response time of 340 s, as the temperature increased from room temperature to 100 °C, while GNP-0 possessed the slowest response time of 500 s. This could be attributed to the increase of the thermal and electrical conductivities by the incorporation of GNPs. A similar trend could be observed for the resistance change of the hybrid films, which indicated that the temperature and resistance changes were significantly synchronized.

### 4.2. Liquid Sensor 

The adsorption–desorption experimental tests depicted that the MWCNT/GNP hybrid film exhibited a highly porous structure, as shown in Figure 8, facilitating easy penetration of electrolyte and a fast transfer of the organic molecules. Thus, it could be potentially used in sensor applications to detect the leakage of organic solvents.

**Figure 14:**
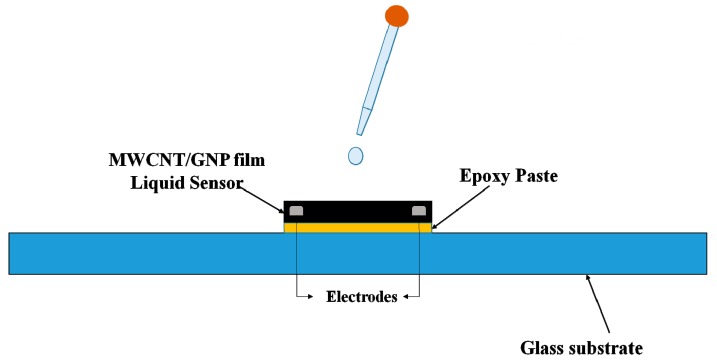
Schematic diagram of the titration test on the hybrid film.

The hybrid film samples were cut into rectangular strips (30 mm × 20 mm) and placed at the center of a glass substrate. Two electrodes were adhered to the hybrid film sensor using a silver paste and measurements were made using a digital multimeter (Voltage source: 0.3 V, Current Rang: 0~25 mA; Keithley 2450). A layer of epoxy adhesive was applied onto the conductive silver paste to provide the protection. Three different organic solvents, namely, isopropanol, methanol, and acetone, were used in the titration test. In this work, five different amounts of organic solvents, ranging from 20 μL to 100 μL (controlled by a pipette), were dropped onto the hybrid film. Figure 14 illustrates the schematic diagram of the titration test.

**Figure 15:**
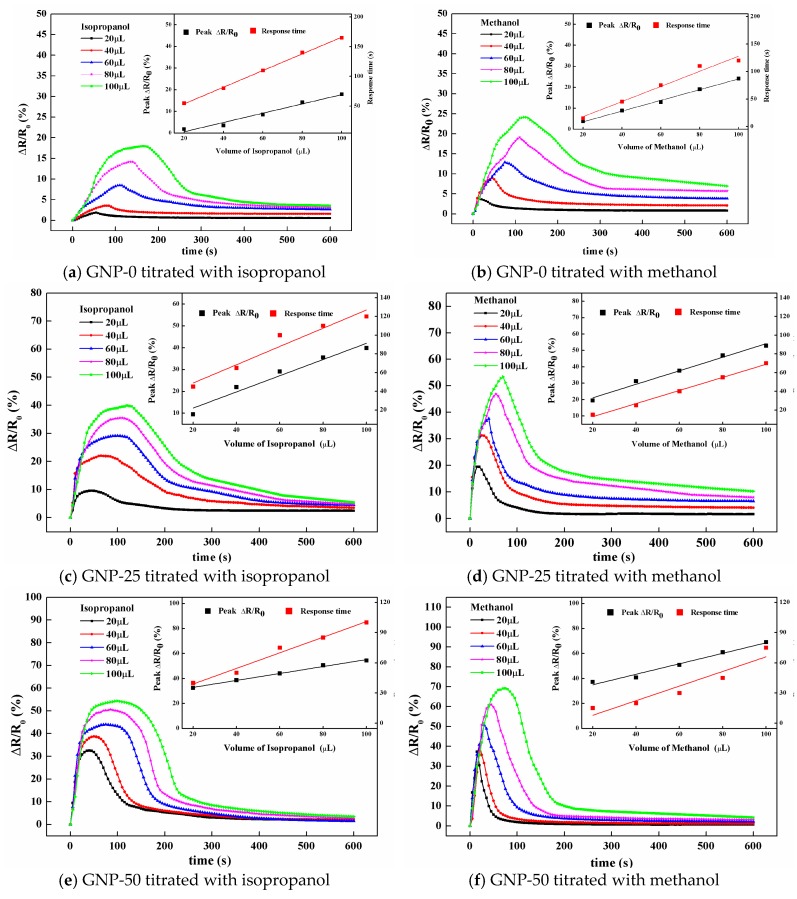
Resistance changes of the hybrid films titrated with different amounts of isopropanol and methanol.

Figure 15 plots the resistance changes of the hybrid films titrated with different amounts of isopropanol and methanol solvents. All the curves demonstrated a similar pattern. Li et al. [8] reported a similar curve for carbon nanotube buckypapers. The resistance response of the hybrid film could be divided into two stages, namely, infiltration and evaporation. In the first stage, the resistance increased with time, until it reached the peak value, which corresponded to the infiltration of the solvent. Upon infiltration, the penetration of the small solvent molecules between the MWCNTs and GNPs, due to the strong capillary effect, formed at the MWCNT-molecule-GNP junctions, which induced the barrier to be tunneled through solvent molecules, for conduction. Furthermore, the distance between the MWCNTs and GNPs increased, resulting in an increase of the tunneling barrier. Thus, a significant increase of the resistance was observed. In the second stage, the solvents were evaporated, the barriers for tunneling conduction, through the MWCNT-molecule-GNP junctions, were reduced. The resistance was gradually recovered. The peak value of the resistance changed and the time to reach the peak are presented in the inset of Figure 15. It appears that both the maximum resistance change and time to reach the maximum linearly increased with an increase in the amount of solvent infiltrated into the film. The resistance change of the hybrid film infiltrated with methanol was larger than that of isopropanol, while the response time was less than that of isopropanol. This could be attributed to the low conductivity and viscosity of the methanol, in comparison with isopropanol. The resistance change and response time were relative to the conductivity and viscosity of the organic solvent, respectively.

**Figure 16:**
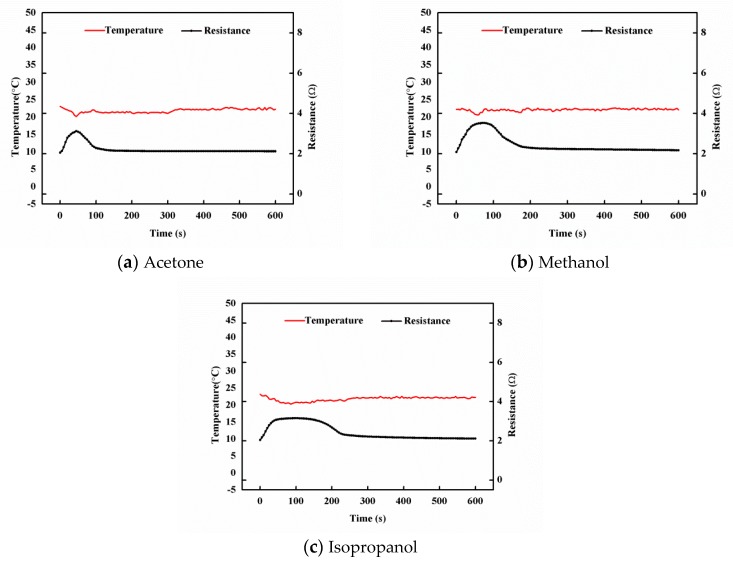
Temperature and resistance responses of the hybrid film GNP-50 titrated with 100 μL of acetone, methanol, and isopropanol.

The temperature variations of the hybrid film, during the titration of the organic solvent, were measured. Figure 16a–c plot the temperature and resistance responses of the hybrid film GNP-50, titrated with 100 μL of acetone, methanol, and isopropanol, respectively. The maximum temperature changes of the hybrid film titrated with acetone, methanol, and isopropanol were 2.9 °C, 3.1 °C, and 2.8 °C, respectively. The effect of the small variation of the temperature on the resistance change, during infiltration, can be neglected. Thus, the resistance change was mainly due to the penetration of small solvent molecules into the hybrid film.

**Figure 17:**
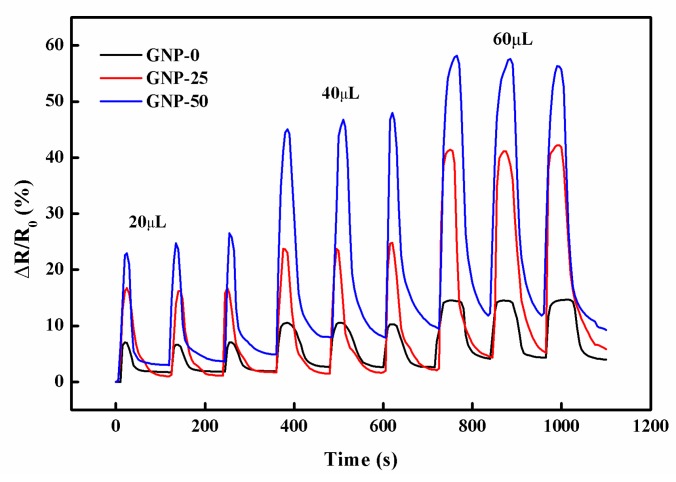
Resistance changes of the hybrid films in consecutive cycles of infiltration and evaporation of acetone drops.

To explore the repeatability and stability of leakage sensing, a series of titration tests were conducted by dropping acetone onto the surface of the hybrid films. Figure 17 shows the resistance changes of the hybrid films in nine consecutive cycles of infiltration and evaporation of acetone drops. The volume of each drop was gradually increased as—20 μL/drop for the first three drops, 40 μL/drop for the second three drops, and 60 μL/drop for the third three drops. It can be observed that the patterns of resistance changes were identical. This demonstrates the high durability and feasibility of the hybrid film as a liquid sensor, to detect both the occurrence and the amount of organic solvent leakage.

## 5. Conclusions

Freestanding MWCNT/GNP hybrid films, with various GNP contents, were successfully prepared by vacuum filtration. Surface and cross-view SEM images showed that MWCNTs and GNPs assembled to form a densely packed film, with a “sandwich”-like structure. A hybrid film with 50 wt% of GNP had a more closely packed arrangement, lower porosity, and a higher density than that of the neat MWCNT film. The thermal conductivity of the hybrid film increased from 168.22 W/m∙k to 364.24 W/m∙k, as the GNP loading increased from 0 to 50 wt%. Nanoindentation tests showed that both the hardness and elastic modulus of the hybrid film decreased with the increase of the GNP content. In this work, the hybrid films were used as temperature and liquid sensors, to monitor the temperature and liquid leakage. It was found that the hybrid film exhibited a negative temperature coefficient of resistance (TCR). The temperature sensitivity of the hybrid film decreased with the increase of the GNP content. The resistance responses of the hybrid film to the organic solvents, including isopropanol, methanol, and acetone, from infiltration and evaporation, were investigated. Experimental results showed that the hybrid film can be used to detect the temperature with humidity insensitivity. Moreover, the hybrid film was capable of detecting the liquid leakage, while the effect of the small variation of the temperature, during the infiltration, could be neglected. The characteristic of the resistance responses demonstrated the capability of the MWCNT/GNP hybrid film for applications in sensing temperature and leakage of organic solvents.

## Figures and Tables

**Table 1:** Thermal properties of the hybrid films with the different weight percentages of GNP.

GNP (wt%)	ρ (g/cm^3^)	Cp (J/g∙k)	α (m^2^/s)	K (W/m∙k)
0	0.685 ± 0.004	1.016 ± 0.003	(2.417 ± 0.179) × 10^−4^	168.16 ± 12.12
25	0.699 ± 0.009	1.032 ± 0.004	(3.371 ± 0.228) × 10^−4^	243.02 ± 12.82
50	0.721 ± 0.009	1.052 ± 0.005	(4.802 ± 0.210) × 10^−4^	364.33 ± 14.36
